# Use of Electronic Nicotine Delivery Systems and Age of Asthma Onset Among US Adults and Youths

**DOI:** 10.1001/jamanetworkopen.2024.10740

**Published:** 2024-05-17

**Authors:** Adriana Pérez, Sarah Valencia, Pushan P. Jani, Melissa B. Harrell

**Affiliations:** 1 Department of Biostatistics and Data Science, The University of Texas Health Science Center at Houston, School of Public Health, Austin; 2 Michael and Susan Dell Center for Healthy Living, The University of Texas Health Science Center at Houston, School of Public Health, Austin; 3 Division of Pulmonary and Sleep Medicine, The University of Texas Health Science Center at Houston, School of Medicine, Houston; 4 Department of Epidemiology, The University of Texas Health Science Center at Houston, School of Public Health, Austin

## Abstract

**Importance:**

The association of use of electronic nicotine delivery systems (ENDS) with the age of asthma onset is unknown.

**Objective:**

To explore the association of past 30-day ENDS use with the age of asthma onset in adults and youths who did not have asthma or chronic obstructive pulmonary disease and never used cigarettes.

**Design, Setting, and Participants:**

This cohort study was a secondary analysis of waves 1 to 6 of the US nationally representative Population of Tobacco and Health Study (2013-2021). Eligible participants included adults (≥18 years) and youths (12-17 years) who did not have asthma or chronic obstructive pulmonary disease at the first wave of participation. Data analysis was conducted from September 2022 to April 2024.

**Exposure:**

Past 30-day ENDS use at the first wave of participation in the study preceding the onset of asthma.

**Main outcome and measures:**

Lower and upper age limits were estimated using the age reported at the first wave of participation and the number of weeks between follow-up waves until asthma was first reported or censored. The association of past 30-day ENDS use with the age of asthma onset was estimated using weighted interval-censoring Cox regression. The cumulative hazard function for the age of asthma onset was estimated using interval-censoring survival analysis.

**Results:**

A total of 24 789 participants were included, with 7766 adults (4461 female [weighted percentage, 59.11%] and 3305 male [weighted percentage, 40.89%]), representing 80.0 million adults, and 17 023 youths (8514 female [weighted percentage, 50.60%] and 8496 male [weighted percentage 49.32%]), representing 33.9 million youths. By age 27 years, 6.2 per 1000 adults reported asthma incidence (hazard ratio [HR], 0.62%; 95% CI, 0.46%-0.75%). While controlling for covariates, there was a 252% increased risk of the onset of asthma at earlier ages for adults who used ENDS in the past 30 days vs adults who did not (adjusted HR, 3.52; 95% CI, 1.24-10.02). For youths, there was no association of ENDS use in the past 30 days with age of asthma onset (adjusted HR, 1.79; 95% CI, 0.67-4.77), which could be due to a lack of statistical power.

**Conclusion and relevance:**

In this cohort study, past 30-day ENDS use among adults was associated with earlier ages of asthma onset. These findings suggest that prevention and cessation programs directed to adults who use ENDS are needed to educate the public, protect public health, prevent adverse health outcomes, and motivate users to stop. Furthermore, modifying symptom-screening asthma guidelines, resulting in earlier asthma detection and treatment, may reduce morbidity and mortality due to asthma.

## Key Points

**Question:**

Is the use of electronic nicotine delivery systems (ENDS) in the past 30 days associated with the age of asthma onset among adults (≥18 years) and youths (12-17 years) who never used cigarettes?

**Findings:**

This cohort study of 7766 adults and 17 023 youths from the Population Assessment of Tobacco and Health Study (2013-2021) found that adults who used ENDS in the past 30-days had an increased risk for asthma incidence at earlier ages vs adults who did not use ENDS in the past 30 days. This finding did not hold for youths.

**Meaning:**

These findings suggest that tobacco regulations, prevention, intervention campaigns, and cessation programs are needed to prevent early age of asthma onset among adults who use ENDS, to protect public health, and to help improve asthma screening guidelines.

## Teaser

This cohort study analyzes the use of electronic nicotine delivery systems and the age of asthma onset among adults and youths in the US.

## Introduction

Asthma is one of the most prevalent respiratory diseases, affecting more than 27 million people in the US (8.4% of adults aged ≥18 years and 5.8% of youths aged 12-17 years).^1,2^ The number of US emergency department visits in 2021 attributable to asthma without chronic obstructive pulmonary disease (COPD) was approximately 939 000 (SE, 149 000), which was 0.7% of all emergency department visits in 2021.^3^ Asthma is one of the most costly diseases, resulting in $300 billion annually in losses due to missed school or work days, mortality, and medical costs,^4,5^ and an estimated total burden of $963.1 billion annually.^6^

Cigarette or combustible product use is associated with increased risk for the onset of asthma.^7^ Electronic nicotine delivery systems (ENDS; ie, vapes, vaporizers, vape pens, hookah pens, electronic cigarettes [e-cigarettes], and/or electronic pipes [e-pipes]) are highly popular in the US. A nationally representative study^8^ found that in 2021 (most recent data available), among adults aged 18 years or older, the prevalence of ENDS use every day or some days was 4.5% (representing 11.1 million adults), with men reporting higher prevalence of ENDS use (5.1%) in comparison with women (4.0%). In 2022, among middle and high school students, the prevalence of past 30-day (P30D) ENDS use was 9.4% (2.55 million students), and the prevalence of P30D ENDS use was 5.2% among non-Hispanic White students, 2.4% among non-Hispanic Black or African American students, 3.3% among Hispanic students, 2.9% among non-Hispanic Asian students, 8.9% among non-Hispanic students of other races and ethnicities (eg, non-Hispanic American Indian or Alaska Native, multiracial individuals, and any other race or ethnicity).^9,10^ Harmful chemical ingredients found in ENDS have been found to affect pulmonary function^11^ and may have the potential to affect respiratory health.^12,13,14,15^ Previous studies have examined the differences by biological sex of the age of asthma onset in adults^16,17,18^ and youths,^19,20^ but not with recent data. Several studies,^21,22,23,24^ but not all,^25,26^ have reported that ENDS use is associated with increased risk of asthma in adults^21,22^ and youths^23,24^ after controlling for other tobacco product (TP) use. While these studies have merit, there is a substantial gap in the field because prior studies have not examined the age of asthma onset with recent data or the association of P30D ENDS use with age of asthma onset for adults and for youths. Quantifying the potential risk of earlier asthma onset due to P30D ENDS use can help prevent people from initiating use or motivate them to stop.

## Methods

### Study Design and Included Participants

This cohort study was approved by the Committee for the Protection of Human Subjects at the University of Texas Health Science Center at Houston and follows the Strengthening the Reporting of Observational Studies in Epidemiology (STROBE) reporting guidelines for cohort studies.^27^ Secondary longitudinal data analysis of restricted data sets^28^ (waves 1-6) of the Population Assessment of Tobacco and Health (PATH) Study^29^ were conducted. This study is a nationally representative, longitudinal cohort study that was designed to measure tobacco use behaviors and how they affect the health of adults (aged ≥18 years) and youths (aged 12-17 years) in the US using a complex design.^29^ Wave 1 was collected in 2013 to 2014, wave 2 in 2014 to 2015, wave 3 in 2015 to 2016, wave 4 in 2016 to 2017, wave 4.5 in 2017 to 2018, wave 5 in 2018 to 2019, wave 5.5 in 2019 to 2020, and wave 6 in 2020 to 2021. The sample size of the wave 1 cohort was 32 320 adults and 13 651 youths.^28^ After wave 3, the PATH Study added a new cohort of participants, including 6065 adults and 3739 youths due to attrition that was referred as the wave 4 cohort.^28^After wave 4, adults were followed-up every 2 years, but youths were followed-up annually. Youths from the wave 1 and wave 4 cohorts were measured in 2017 and 2018 as wave 4.5. In addition, at wave 1, a shadow youth sample of 9- to 11-year-olds was collected to replenish the 12- to 17-year-olds who became adults (ie, turned 18 years of age) in waves 2 to 5.5. There were 2091 shadow youths who entered the study at wave 2, 2045 at wave 3, 1694 at wave 4, 1753 at wave 4.5, 1652 at wave 5, and 53 at wave 5.5.^28^ There were 1915 youths who turned 18 in wave 1 and participated in the adult survey at wave 2, 1907 who turned 18 in wave 2 and participated in the adult survey at wave 3, 1900 who turned 18 in wave 3 and participated in the adult survey at wave 4, 4443 who turned 18 in wave 4 and participated in the adult survey at wave 5, 2614 who turned 18 in wave 5 and participated in the adult survey at wave 5.5, and 53 who turned 18 in wave 5.5 and participated in the adult survey at wave 6.^28^ Adults older than 20 years in 2020 were measured using an adult telephone survey and their data were also included to evaluate the age of asthma onset. The analysis included adults who entered the PATH study at wave 1 and wave 4; these cohorts were tracked longitudinally across waves after entry.^28^ Similarly, the analysis for youths included those who entered the PATH study at wave 1 and wave 4. In addition, shadow youths who entered at each wave (as replacements) were appended to these cohorts. Finally, wave 1, wave 4, and shadow youths who turned 18 at any wave were invited to complete the adult measurements thereafter and were tracked longitudinally.^28^ The reasons for excluding participants and number and percentage of participants excluded or included in these analyses are detailed in eTable 1 in Supplement 1. All youth participants provided informed written assent and parents provided informed written consent for youths. Adult participants provided informed written consent.

### Measures

#### Asthma and COPD Assessment

Adult participants were asked at their first wave of adult participation (waves 1-6), “Has a doctor or other health professional ever told you that you had [asthma] [COPD]?” Adults who answered no at their first wave of adult participation were asked about asthma diagnosis in the past 12 months in waves 2 through 6. Parents of youths participated in the study to enhance the accuracy of some questions. At the youths’ first wave of study participation, parents of youths were asked, “Has [Child] ever been told by a doctor or other health professional that [he/she] has asthma?” COPD was not measured in youths. Youths with no asthma at their first wave of study participation (waves 1-5.5) were asked about asthma diagnosis in the past 12 months in waves 2 to 6. Age of asthma onset was estimated using waves 2 to 6 (includes waves 4.5 [youths only], 5.5 [youths and adults], and the adult telephone survey [adults only]).

#### P30D ENDS Use

In waves 1 and 2, P30D ENDS use was measured with the question, “In the past 30 days, have you used an e-cigarette, even one or two times?” and in waves 3 to 5.5 with the question, “In the past 30 days, have you used an electronic nicotine product, even one or two times? Electronic nicotine products include e-cigarettes, vape pens, personal vaporizers and mods, e-cigars, e-pipes, e-hookahs, and hookah pens,” assuming they were the same construct. P30D ENDS use was estimated at the first wave of participation (2013-2020), which happened before asthma incidence (2014-2021).

#### Covariates at First Wave of Participation

The PATH Study imputed sex, race, and Hispanic ethnicity using the household information.^28^ Adults self-reported their sex, race, and Hispanic ethnicity and parents reported sex, race, and Hispanic ethnicity for youths. Race was categorized as Asian, Black, White, and other race (ie, multiracial and any other race not otherwise specified). Ethnicity was categorized as either Hispanic or non-Hispanic. Answers to race and ethnicity questions were combined to create race and ethnicity categories including Hispanic, non-Hispanic Black, non-Hispanic White, and other (non-Hispanic Asian, multiracial, and any other race or ethnicity not otherwise specified). Race and ethnicity were included to account for disparities. Other covariates included adults’ education or education of youths’ parents (collapsed as less than high school, high school or general education development test, some college or associate’s degree, and bachelor’s degree or higher), binge drinking (male adults, ≥5 drinks; female adults, ≥4 drinks; boys aged 9-13 years, ≥3 drinks; boys aged 14-15 years, ≥4 drinks; boys aged 16-17 years, ≥5 drinks; girls, ≥3 drinks, each one in a single sit),^30^ ever using marijuana,^31^ total number of years that a participant reported ENDS use (subtraction of the age when the participant first reported using ENDS from the age at the first wave of participation), participant’s P30D use of other TP (eg, filtered cigars, traditional cigars, cigarillos, hookah, and smokeless tobacco), total number of waves of emergent cigarette use prior to asthma onset, any person who uses TPs present at home,^32^ rules at home about any TP use (never allowed vs allowed anywhere, sometimes, or anytime),^33^ and weight status.^34^

#### Age of Asthma Onset Estimation

Because the exact date of asthma onset was not assessed and the participants’ birthdays were not provided, the age at the first wave of study participation and the number of weeks between survey dates were used to estimate the age of asthma onset prospectively. Lower and upper age bounds for asthma onset were estimated for the time-to-event analyses. The lower age bound was the age at the last wave where participants did not report asthma and the upper age bound was age at the wave that participant reported asthma incidence. Participants who reported no asthma in waves 1 to 6 were censored. The age of asthma onset was estimated to occur between the lower and upper age bounds in all participants.

### Statistical Analysis

Statistical analysis was conducted from September 2022 to April 2024. All statistical analyses incorporated the use of sampling weights and 100 balance repeated replicate weights at the first wave of study participation with the Fay adjustment set to 0.3.^28^ Weighted summary statistics were reported to describe sociodemographic characteristics, prevalence of P30D ENDS use, other TP use, and other covariates at participants’ first wave of study participation. The median follow-up time was estimated by adding the number of weeks between the first wave of participation and (1) the first wave of asthma onset or (2) the last wave of participation (waves 2-6) for censored participants. For participants who were lost to follow-up from the first wave of participation to wave 6, a week of participation was assumed to estimate the median follow-up time. Weighted interval-censoring survival analyses were implemented for adults and youths to estimate the age of asthma onset and are presented as graphs.^35,36,37,38^ The association of P30D ENDS use with the age of asthma onset was explored by fitting weighted interval-censoring Cox proportional hazards models using cubic splines (3 knots)^39,40^ as the baseline hazard function.^41^ Hazard ratios (HRs) and 95% CIs were reported. Sociodemographic characteristics and other TP use were included in final models regardless of significance. Three models were fit. Model 1 included covariates with a 2-sided *P* value < .20 in the univariate analysis. Model 2 removed covariates that were not significant from model 1. Model 3 included the covariates in model 2, total number of years that a participant reported ENDS use, and ever marijuana use. The threshold for statistical significance for models 2 and 3 was a type I error level of .05. Statistical analyses were conducted in SAS version 9.4-TSlevel1M6 (SAS Institute).

One sensitivity analysis was conducted for adults and youths who reported not having asthma or COPD regardless of their TP use. For the sensitivity analyses, the reasons for exclusion and number and percentage of participants excluded or included are detailed in eTable 2 in Supplement 1. Combustible TP use (ie, never, former, and P30D) and P30D ENDS use were combined in 6 mutually exclusive categories using the never use category of combustible TP and no P30D ENDS use as the reference category (P30D combustible TP use and P30D ENDS use status). At the first wave of participation, the lifetime pack-years of cigarette use was estimated using the same methodology as in previous publications^42^ as a potential covariate. Only models 1 and 2 were fitted.

## Results

The total sample size for analysis was 24 789 at first wave of participation and included 7766 adults (4461 female [weighted percentage, 59.11%] and 3305 male [weighted percentage, 40.89%]), representing approximately 80.0 million adults, and 17 023 youths (8514 female [weighted percentage, 50.60%] and 8496 male [weighted percentage 49.32%]), representing approximately 33.9 million youths. Table 1 shows the demographic characteristics of adults and youths that did not have asthma or COPD and who never used cigarettes at their first wave of participation. Among adults, 1765 were Hispanic (weighted percentage, 19.62%), 1534 were non-Hispanic Black (weighted percentage, 14.42%), 3712 were non-Hispanic White (weighted percentage, 54.19%), and 755 were other race or ethnicity (weighted percentage, 11.77%). Among youths, 4944 were Hispanic (weighted percentage, 23.67%), 2128 were non-Hispanic Black (12.76%), 8334 were non-Hispanic White (weighted percentage, 52.95%), and 1584 were other race or ethnicity (weighted percentage 10.40%). The median (SE) follow-up time in the study was 4.94 (0.06) years among adults and 4.19 (0.04) years among youths.

**Table 1.** Demographic and Measure Characteristics of Adults and Youth That Reported Not Having Asthma or Chronic Obstructive Pulmonary Disease and Never Using Cigarettes in the PATH Study (2013-2020) at the First Wave of Participation^a^

Variable reported at the first wave of participation in the PATH Study^a^	Adults			Youths		
	Sample No. (n = 7766)^b^	Estimated national population No. (N = 80 006 590)^b^	Weighted % (SE)	Sample No. (n = 17 023)^b^	Estimated national population No. (N = 33 901 949)^b^	Weighted % (SE)
Wave of entry into the PATH Study^a^						
1 (2013-2014)	6197	68 787 579	85.98 (0.42)	9572	17 499 009	51.62 (0.22)
2 (2014-2015)	NA	NA	NA	1641	3 250 569	9.59 (0.13)
3 (2015-2016)	NA	NA	NA	1629	3 376 660	9.96 (0.15)
4 (2016-2017)	1569	11 219 011	14.02 (0.42)	1352	2 484 943	7.33 (0.11)
4.5 (2017-2018)	NA	NA	NA	1406	3 463 614	10.22 (0.15)
5 (2018-2019)	NA	NA	NA	1358	3 570 838	10.53 (0.15)
5.5 (2019-2020)	NA	NA	NA	43	159 405	0.47 (0.07)
6 (2020-2021)	NA	NA	NA	22	96 910	0.29 (0.07)
Age, weighted mean (SE), y	NA	NA	43.64 (0.27)	NA	NA	13.25 (0.01)
Sex						
Female	4461	47 289 462	59.11 (0.62)	8514	17 154 101	50.60 (0.20)
Male	3305	32 717 128	40.89 (0.62)	8496	16 719 119	49.32 (0.20)
Missing	NA	NA	NA	13	28 728	0.08 (0.02)
Race and ethnicity						
Hispanic	1765	15 696 967	19.62 (0.46)	4944	8 024 808	23.67 (0.19)
Non-Hispanic Black	1534	11 534 680	14.42 (0.40)	2128	4 325 250	12.76 (0.18)
Non-Hispanic White	3712	43 356 352	54.19 (0.77)	8334	17 952 377	52.95 (0.29)
Other^c^	755	9 418 592	11.77 (0.38)	1584	3 526 074	10.40 (0.19)
Missing	NA	NA	NA	33	73 441	0.22 (0.05)
Education level^d^						
Less than high school	1149	12 063 137	15.08 (0.46)	3307	5 630 413	16.61 (0.42)
High school or general educational development test	2061	20 054 429	25.07 (0.55)	3009	5 723 448	16.88 (0.34)
Some college or associate’s degree	2662	23 346 682	29.18 (0.57)	6653	13 819 598	40.76 (0.54)
Bachelor’s degree or higher	1894	24 542 343	30.68 (0.57)	3865	8 362 711	24.67 (0.70)
Missing	NA	NA	NA	189	365 778	1.08 (0.07)
Weight status^e^						
Underweight	293	2 665 443	3.33 (0.27)	850	1 754 907	5.18 (0.18)
Healthy weight	3062	27 794 107	34.74 (0.63)	10 400	21 053 211	62.10 (0.42)
Overweight	2224	24 822 350	31.03 (0.69)	2707	5 241 997	15.46 (0.31)
Obesity class 1 adult	1170	12 938 049	16.17 (0.55)	1561	2 939 576	8.67 (0.23)
Obesity class 2 adult	816	9 338 905	11.67 (0.47)			
Severe obesity (youth)	NA	NA	NA	728	1 335 179	3.94 (0.17)
Missing	201	2 447 736	3.06 (0.24)	777	1 577 079	4.65 (0.17)
Past 30-d ENDS use^f^						
No	7606	79 487 772	99.35 (0.08)	16 927	33 711 816	99.44 (0.07)
Yes	160	518 818	0.65 (0.08)	96	190 133	0.56 (0.07)
Past 30-d any cigar product use						
No	7438	78 401 806	97.99 (0.18)	16 797	33 459 208	98.69 (0.11)
Yes	292	1 018 530	1.27 (0.09)	50	97 486	0.29 (0.05)
Missing	36	586 254	0.73 (0.14)	176	345 255	1.02 (0.08)
Past 30-d hookah use						
No	7483	78 974 511	98.71 (0.11)	16 920	33 708 410	99.43 (0.08)
Yes	270	843 644	1.05 (0.09)	68	128 397	0.38 (0.07)
Missing	13	188 435	0.24 (0.08)	35	65 142	0.19 (0.04)
Past 30-d smokeless tobacco use						
No	7530	78 981 405	98.72 (0.10)	16 838	33 553 392	98.97 (0.08)
Yes	221	870 978	1.09 (0.07)	30	63 951	0.19 (0.03)
Missing	15	154 207	0.19 (0.06)	155	284 606	0.84 (0.07)
Total years of reported ENDS use, No.^f^						
0	7372	78 675 358	98.34 (0.11)	16 795	33 463 454	98.71 (0.11)
1	180	571 886	0.71 (0.07)	181	351 003	1.04 (0.10)
≥2	198	698 576	0.87 (0.08)	43	80 229	0.24 (0.04)
Missing	16	60 770	0.08 (0.02)	4	7263	0.02 (0.01)
Binge drinking						
No	7336	77 410 007	96.75 (0.25)	16 803	33 466 229	98.71 (0.11)
Yes	403	2 293 462	2.87 (0.23)	97	181 372	0.54 (0.06)
Missing	27	303 121	0.38 (0.10)	123	254 348	0.75 (0.08)
Ever marijuana use						
No	6403	71 391 858	89.23 (0.43)	16 333	32 622 387	96.23 (0.16)
Yes	1335	8 379 375	10.47 (0.42)	604	1 097 304	3.24 (0.14)
Missing	28	235 358	0.29 (0.07)	86	182 258	0.54 (0.07)
Any person who uses tobacco product present at home						
No	6122	67 298 662	84.12 (0.65)	11 658	23 500 518	69.32 (0.67)
Yes	1617	12 387 159	15.48 (0.64)	5128	9 958 052	29.37 (0.66)
Missing	27	320 769	0.40 (0.09)	237	443 378	1.31 (0.09)
Rules at home about any tobacco product use (anywhere, sometimes, or anytime)						
Never allowed	6144	66 283 355	82.84 (0.70)	12 488	24 904 772	73.46 (0.53)
Allowed	1606	13 470 160	16.84 (0.69)	4378	8 679 744	25.60 (0.53)
Missing	16	253 076	0.32 (0.10)	157	317 433	0.94 (0.07)

Abbreviations: ENDS, electronic nicotine delivery systems; NA, not applicable; PATH, Population Assessment of Tobacco and Health Study.

^a^ The restricted file received disclosure to publish between October 25, 2023, and April 10, 2024.^28^

^b^ Any difference in categories of response with the total sum of the estimated population size is due to rounding of decimals.

^c^ Other included Non-Hispanic Asian, multiracial, and any other race or ethnicity not otherwise specified.

^d^ Highest level of education attained by participants for adults or highest level of education attained by parents for youths.

^e^ Weight status was calculated as follows: Body mass index (BMI) was calculated as weight in kilograms divided by height in meters squared. Adults with a BMI score less than 18.5 were classified as underweight, 18.5 to 25 as healthy weight, 25 to 30 as overweight, 30 to 35 as obesity class 1, and greater than 35 as obesity class 2. Youth BMI was calculated using the Centers for Disease Control BMI-for-age percentile growth charts that utilize the child’s age, height, and weight to determine percentiles. Youths who had a BMI less than the 5th percentile were classified as underweight, between the 5th and less than the 85th percentile as healthy weight, between the 85th to 95th percentile as overweight, greater than the 95th percentile or greater as obese, and 120% of the 95th percentile or greater or BMI of 35 or greater as severe obesity.

^f^ ENDS include vapes, vaporizers, vape pens, hookah pens, electronic cigarettes, and/or electronic pipes.

The Figure shows the distribution of the estimated age of asthma onset. We found that among adults who did not have asthma or COPD and who never used cigarettes, 6.2 per 1000 adults reported incidence of asthma by age 27 years (HR, 0.62%; 95% CI, 0.46%-0.75%). By age 35 years, 10.8 per 1000 adults reported incidence of asthma (HR, 1.08%; 95% CI, 0.82%-1.42%), and by age 55 years, 25.8 per 1000 adults reported incidence of asthma (HR, 2.58%; 95% CI, 2.09%-3.24%). Also, we found that 22.3 per 1000 youths reported incidence of asthma by age 15 years (HR, 2.23%; 95% CI, 1.88%-2.45%) and 79.6 per 1000 youths reported incidence of asthma by age 20 years (HR, 7.96%; 95% CI, 7.31%-8.61%).

**Figure.**
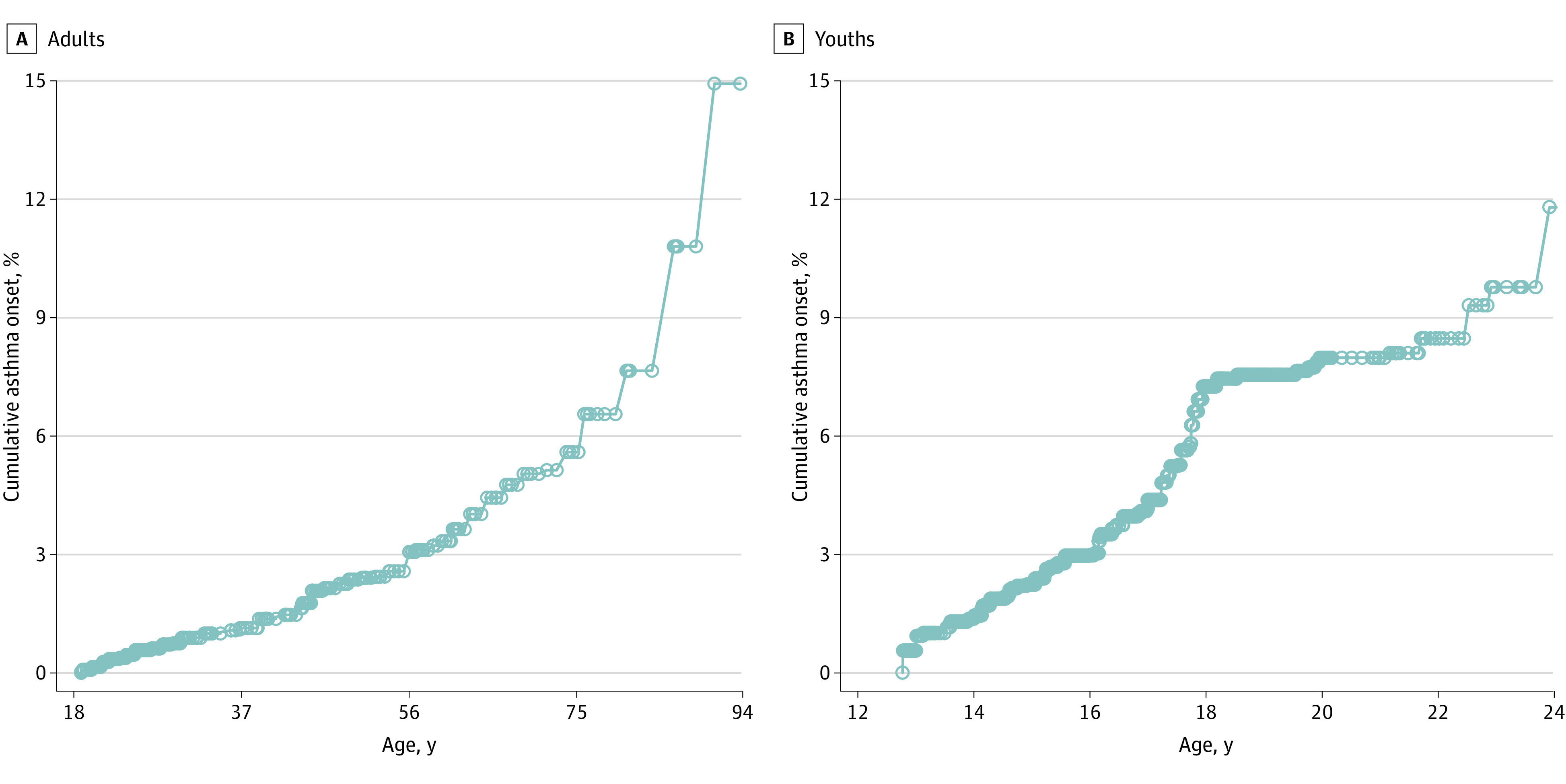
Age of Asthma Onset Among Participants Who Did Not Have Asthma or Chronic Obstructive Pulmonary Disease and Never Used Cigarettes at the First Wave of Participation in the Population Assessment of Tobacco and Health (PATH) Study, 2013-2021 The cumulative hazard function is shown for adults (A) and youths (B).

Table 2 shows the crude and adjusted Cox proportional hazard models for adults. Model 1 shows that after adjusting for covariates, adults who with P30D ENDS use were at a 252% increased risk of asthma onset at earlier ages compared with adults who reported no P30D ENDS use (HR 3.52, 95% CI 1.24-10.02). When reducing the number of covariates in models 2 and 3, the results were similar.

**Table 2.** Association of Past 30-Day ENDS Use With the Age of Asthma Onset Among the 7766 Adults Who Did Not Have Asthma or Chronic Obstructive Pulmonary Disease Free and Never Used Cigarettes at the First Wave of Participation in the PATH Study, 2013-2021^a^

Variable reported at the first wave of participation in the PATH Study^a^	Crude HR (95% CI)	Adjusted HR (95% CI)		
		Model 1	Model 2	Model 3
Past 30-d ENDS use^b^				
No	1[Reference]	1 [Reference]	1 [Reference]	1 [Reference]
Yes	9.57 (3.76-24.33)	3.52 (1.24-10.02)	3.70 (1.24-11.05)	3.66 (1.23-10.85)
Sex				
Male	1 [Reference]	1 [Reference]	1 [Reference]	1 [Reference]
Female	1.10 (0.78-1.56)	1.30 (0.89-1.89)	1.31 (0.90-1.92)	1.31 (0.90-1.93)
Race and ethnicity				
Hispanic	0.86 (0.54-1.36)	1.08 (0.63-1.86)	1.05 (0.60-1.82)	1.04 (0.60-1.81)
Non-Hispanic Black	1.47 (0.99-2.18)	1.39 (0.92-2.11)	1.40 (0.93-2.11)	1.40 (0.93-2.11)
Non-Hispanic White	1[Reference]	1[Reference]	1[Reference]	1[Reference]
Other^c^	0.91 (0.40-2.07)	0.84 (0.45-1.59)	0.84 (0.45-1.58)	0.84 (0.45-1.58)
Education				
Bachelor’s degree or higher	1 [Reference]	1 [Reference]	1 [Reference]	1 [Reference]
Less than high school	0.79 (0.46-1.34)	0.67 (0.34-1.32)	0.71 (0.36-1.39)	0.71 (0.36-1.40)
High school or general educational development test	1.03 (0.61-1.74)	0.91 (0.55-1.50)	0.96 (0.59-1.57)	0.96 (0.58-1.57)
Some college or associate’s degree	1.32 (0.82-2.11)	1.15 (0.71-1.85)	1.18 (0.73-1.91)	1.18 (0.73-1.91)
Weight status^d^				
Healthy weight	1 [Reference]	1 [Reference]	1 [Reference]	1 [Reference]
Underweight	0.61 (0.23-1.63)	0.60 (0.21-1.68)	0.64 (0.23-1.78)	0.64 (0.23-1.78)
Overweight	1.03 (0.70-1.52)	0.99 (0.65-1.52)	0.99 (0.65-1.51)	0.99 (0.65-1.51)
Obesity class 1	1.06 (0.62-1.79)	1.02 (0.61-1.73)	1.04 (0.62-1.76)	1.05 (0.62-1.76)
Obesity class 2	1.83 (1.10-3.05)	1.61 (0.91-2.86)	1.70 (0.98-2.96)	1.70 (0.98-2.96)
Past 30-d any cigar product use^e^				
No	1 [Reference]	1 [Reference]	1 [Reference]	1 [Reference]
Yes	3.29 (1.70-6.38)	1.64 (0.76-3.54)	1.62 (0.74-3.54)	1.61 (0.72-3.60)
Past 30-d hookah use^e^				
No	1[Reference]	1[Reference]	1[Reference]	1[Reference]
Yes	6.95 (2.99-16.17)	2.23 (0.77-6.44)	2.23 (0.77-6.42)	2.24 (0.74-6.79)
Past 30-d smokeless tobacco use^e^				
No	1 [Reference]	1 [Reference]	1 [Reference]	1 [Reference]
Yes	1.21 (0.31-4.69)	1.04 (0.25-4.38)	1.18 (0.31-4.54)	1.18 (0.31-4.54)
Total years of reported ENDS use, No.^b^				
0	1 [Reference]	1 [Reference]	NA	1 [Reference]
1	2.68 (0.71-10.09)	1.01 (0.17-6.02)	NA	1.09 (0.23-5.08)
≥2	5.40 (1.45-20.12)	1.06 (0.18-6.15)	NA	1.01 (0.16-6.37)
Binge drinking^e^				
No	1 [Reference]	1 [Reference]	1 [Reference]	1 [Reference]
Yes	3.63 (1.51-8.69)	2.37 (0.88-6.37)	2.57 (0.99-6.65)	2.58 (0.99-6.66)
Ever marijuana use^e^				
No	1 [Reference]	1 [Reference]	1 [Reference]	1 [Reference]
Yes	2.59 (1.57-4.27)	2.28 (1.27-4.09)	2.37 (1.34-4.20)	2.37 (1.33-4.21)
Any person who uses tobacco product present at home^e^				
No	1 [Reference]	1 [Reference]	NA	NA
Yes	1.67 (1.12-2.49)	1.31 (0.83-2.09)	NA	NA
Rules at home about any tobacco product use (anywhere, sometimes, or anytime)^e^				
Never allowed	1 [Reference]	1 [Reference]	NA	NA
Allowed	1.53 (1.02-2.30)	1.29 (0.82-2.02)	NA	NA
No. of waves reporting emerging cigarette use prior to asthma onset, weighted mean (SE)	1.19 (0.71-2.00)	0.94 (0.52-1.74)	NA	NA

Abbreviations: ENDS, electronic nicotine delivery systems; HR, hazard ratio; NA, not applicable; PATH, Population Assessment of Tobacco and Health Study.

^a^ The restricted file received disclosure to publish between October 25, 2023, and October 31, 2023.^28^

^b^ ENDS include vapes, vaporizers, vape pens, hookah pens, electronic cigarettes, and/or electronic pipes.

^c^ Other includes Non-Hispanic Asian, multiracial, and any other race or ethnicity not otherwise specified.

^d^ Adult weight status was determined with body mass index, which was calculated as weight in kilograms divided by height in meters squared. Adults with a body mass index less than 18.5 were classified as underweight, 18.5 to 25 as healthy weight, 25 to 30 as overweight, 30 to 35 as obesity class 1, and greater than 35 as obesity class 2.

^e^ The did not respond category is accounted for in the model but the HR is not shown.

Table 3 shows the crude and adjusted Cox proportional hazard models for youths. Model 1 shows that while adjusting for covariates, there was no association of P30D ENDS use with the age of asthma onset (HR, 1.79; 95% CI, 0.67-4.77). When reducing the number of covariates in models 2 and 3, the results were similar. The sensitivity analyses are reported in eTable 3, eTable 4, eTable 5, and the eFigure in Supplement 1. In model 2, when compared with adults who reported never using combustible TP and no P30D ENDS use, adults who reported P30D use of any combustible TP and no P30D ENDS use had a 69% increased risk of earlier ages of asthma onset (HR, 1.69; 95% CI, 1.25-2.29) and adults who reported P30D use of any combustible TP and P30D ENDS use had a 143% increased risk of earlier ages of asthma onset (HR, 2.43; 95% CI 1.71-3.34) ( eTable 4 in Supplement 1). There was no association of the interaction between the P30D use of any combustible TP and P30D ENDS use with the age of asthma onset among youths (eTable 5 in Supplement 1).

**Table 3.** Association of Past 30-Day ENDS Use With the Age of Asthma Onset Among the 17 023 Youths Who Did Not Have Asthma and Never Used Cigarettes at the First Wave of Participation in the PATH Study, 2013-2021^a^

Variable reported at the first wave of participation in the PATH Study^a^	Crude HR (95% CI)	Adjusted HR (95% CI)		
		Model 1	Model 2	Model 3
Past 30-d ENDS use^b^				
No	1 [Reference]	1 [Reference]	1 [Reference]	1 [Reference]
Yes	1.18 (0.46-2.70)	1.79 (0.67-4.77)	1.57 (0.60-4.13)	1.55 (0.60-3.96)
Sex^c^				
Male	1 [Reference]	1 [Reference]	1 [Reference]	1 [Reference]
Female	1.46 (1.27-1.69)	1.44 (1.24-1.67)	1.44 (1.24-1.67)	1.44 (1.24-1.67)
Race and ethnicity^c^				
Hispanic	0.95 (0.83-1.09)	0.97 (0.82-1.16)	0.96 (0.81-1.13)	0.96 (0.81-1.13)
Non-Hispanic Black	1.20 (0.97-1.50)	1.14 (0.91-1.42)	1.13 (0.91-1.42)	1.14 (0.91-1.42)
Non-Hispanic White	1 [Reference]	1 [Reference]	1 [Reference]	1 [Reference]
Other^d^	1.02 (0.82-1.26)	0.99 (0.79-1.23)	0.98 (0.79-1.22)	0.98 (0.79-1.22)
Parent’s education^c^				
Bachelor’s degree or higher	1 [Reference]	1 [Reference]	1 [Reference]	1 [Reference]
Less than high school	0.96 (0.78-1.18)	0.88 (0.68-1.12)	0.88 (0.69-1.12)	0.88 (0.69-1.13)
High school or general educational development test	0.75 (0.59-0.95)	0.68 (0.53-0.87)	0.68 (0.53-0.88)	0.68 (0.53-0.88)
Some college or associate’s degree	0.98 (0.78-1.23)	0.90 (0.71-1.13)	0.90 (0.72-1.14)	0.91 (0.72-1.14)
Weight status^e^				
Healthy weight	1 [Reference]	1 [Reference]	1 [Reference]	1 [Reference]
Underweight	0.72 (0.48-1.09)	0.77 (0.51-1.17)	0.77 (0.51-1.18)	0.77 (0.51-1.18)
Overweight	0.95 (0.76-1.19)	0.98 (0.78-1.24)	0.99 (0.78-1.25)	0.99 (0.78-1.25)
Obesity	1.15 (0.89-1.49)	1.23 (0.95-1.59)	1.23 (0.95-1.59)	1.23 (0.95-1.59)
Severe obesity	1.43 (1.01-2.03)	1.48 (1.02-2.15)	1.50 (1.03-2.17)	1.50 (1.03-2.18)
Past 30-d any cigar product use^c^				
No	1 [Reference]	1 [Reference]	1 [Reference]	1 [Reference]
Yes	0.81 (0.18-3.55)	1.03 (0.22-4.71)	0.98 (0.20-4.70)	1.00 (0.21-4.78)
Past 30-d hookah use^c^				
No	1 [Reference]	1 [Reference]	1 [Reference]	1 [Reference]
Yes	0.70 (0.20-2.40)	0.85 (0.23-3.10)	0.80 (0.22-2.94)	0.76 (0.21-2.80)
Past 30-d smokeless tobacco use^c^				
No	1 [Reference]	1 [Reference]	1 [Reference]	1 [Reference]
Yes	1.01 (0.16-6.22)	1.62 (0.25-10.58)	1.69 (0.26-10.92)	1.67 (0.25-10.98)
Total No. of years that a participant reported ENDS use^b^				
0	1 [Reference]	1 [Reference]	NA	NA
≥1	0.30 (0.10-0.89)	0.36 (0.12-1.05)	NA	NA
Binge drinking^c^				
No	1 [Reference]	1 [Reference]	1 [Reference]	NA
Yes	0.49 (0.12-2.03)	0.59 (0.14-2.51)	0.58 (0.13-2.47)	NA
Ever marijuana use^c^				
No	1 [Reference]	1 [Reference]	1 [Reference]	NA
Yes	0.87 (0.63-1.21)	1.11 (0.76-1.64)	1.08 (0.73-1.59)	NA
Any person who uses tobacco product present at home^c^				
No	1 [Reference]	1 [Reference]	1 [Reference]	1 [Reference]
Yes	1.26 (1.08-1.48)	1.32 (1.09-1.61)	1.35 (1.13-1.62)	1.35 (1.13-1.61)
Rules about tobacco products inside home (anywhere, sometimes, or anytime)^c^				
Not allowed	1 [Reference]	1 [Reference]	NA	NA
Allowed	1.14 (0.99-1.31)	1.08 (0.91-1.29)	NA	NA
No. of waves reporting emerging cigarette use prior to asthma onset, weighted mean (SE)	0.43 (0.32-0.57)	0.42 (0.31-0.56)	0.42 (0.31-0.56)	0.42 (0.31-0.56)

Abbreviations: ENDS, electronic nicotine delivery systems; HR, hazard ratio; NA, not applicable; PATH, Population Assessment of Tobacco and Health Study.

^a^ The restricted file received disclosure to publish between October 25, 2023, and April 10, 2024.^28^

^b^ ENDS include vapes, vaporizers, vape pens, hookah pens, electronic cigarettes, and/or electronic pipes.

^c^ The did not respond category is accounted for in the model but the HR is not shown.

^d^ Other included Non-Hispanic Asian, multiracial, and any other race or ethnicity not otherwise specified.

^e^ Weight status was calculated as follows: Body mass index (BMI) is calculated as weight in kilograms divided by height in meters squared. Youth BMI was calculated using the Centers for Disease Control BMI-for-age percentile growth charts that utilize the child’s age, height, and weight to determine percentiles. Youths who had a BMI less than the 5th percentile were classified as underweight, between the 5th and less than the 85th percentile as healthy weight, between the 85th to 95th percentile as overweight, greater than the 95th percentile or greater as obese, and 120% of the 95th percentile or greater or BMI of 35 or greater as severe obesity.

## Discussion

This cohort study is the first study, to our knowledge, to prospectively estimate the age of asthma onset among adults and youths who never used cigarettes and did not have asthma or COPD at the first wave of participation using interval-censoring survival analyses in a US representative sample. The incidence of asthma onset in adults did not decrease with increasing age. The change in the hazard function increased sharply among those aged older than 60 years. This finding is consistent with a 2005 review^16^ that reported a pooled estimate (including prospective cohorts from Arizona, Michigan, Norway, Sweden, Spain, Poland, Finland, and Denmark) of the adult incidence of asthma as 5.9 per 1000 women (95% CI, 4.4-7.8) and 4.4 per 1000 men (95% CI, 3.1-6.2). These pooled estimates included US data from 1959 to 1996 and are the most recent incidence studies found. Our estimates provide up-to-date information through 2021. Pooled estimates of 29 centers from 14 countries from the European Community Respiratory Health Survey^16^ between 1991 and 2002 reported the adult incidence of asthma by age 35 years as 4.42 (95% CI, 3.22-5.62) per 1000 women and 1.59 (95% CI 0.83-2.34) per 1000 men. Our study included adults who reported never, former, and current P30D cigarette use. We reported the hazard function for the age of onset by age 35 years as 10.8 per 1000 adults, which is almost 2.5 times higher than European countries and twice higher than earlier data from the US.^16^ In youths (those without asthma and those who never used cigarettes), the distribution of the hazard function showed an inflection point at 18 years, which is similar to an observed inflection point on the age of asthma onset seen at 16 years among German youths between 1990 and 2010.^19^ These German children were selected from obstetric departments that were followed up from birth for 20 years with an incidence of asthma of 13.4 per 1000 youths,^19^ which is lower than the 79.6 per 1000 youths in our study. Our results are consistent with previous reports^17,18^ indicating that asthma is increasing in the US and worldwide.

Our results showed that adults who reported never using cigarettes, not having asthma or COPD, and P30D ENDS use at the first wave of participation had increased risk of asthma incidence at earlier ages in comparison with adults who reported no P30D ENDS use. Our sensitivity analysis among adults who did not have asthma or COPD showed a dose response association of P30D tobacco use with earlier age of asthma onset. These findings are in contrast with a 2014 to 2015 study^25^ that started with adults without asthma aged 18 to 39 years and found that use of P30D ENDS or P30D use of any combustible product was not associated with asthma incidence 1 or 2 years later.

While a cross-sectional study^24^ (using 2015-2019 data) reported a significant association of ever using ENDS with asthma among youths who had never used combustible TP, a 2013-2019 study^26^ did not find an association in youths with asthma or respiratory symptoms. This latter finding is consistent with our findings of no association of P30D ENDS use with the age of asthma onset among youths. Our findings may also simply indicate the need for longer follow-up time because asthma onset may take longer time to manifest.

### Strengths and Limitations

The primary strength of our study is the use of the PATH Study^29^ because few studies can test hypotheses with longitudinal data on tobacco-related health outcomes, including asthma, using a nationally representative sample of US adults and youths. Another strength is prospectively estimating the age of asthma onset between 2013 and 2021. This study also has limitations. Other factors associated with age of asthma onset include environmental factors,^43^ nutritional intake,^44,45^ maternal smoking status,^46^ genes,^47,48^ allergies,^49^ dust mites,^50^ and family history of asthma,^51^ but these factors were not measured in the PATH Study, and our results should be cautiously interpreted. The association of other covariates with the age of asthma onset were reported but not discussed because they were selected as covariates. Another limitation is that the PATH Study^29^ did not ask participants the exact date of their asthma diagnosis, so we estimated the age of asthma onset with interval-censoring survival methods. Other researchers may examine the P30D ENDS, daily ENDS use, number of puffs, or first flavor use as a time-varying variable. We limited the analysis to evaluate the association of P30D ENDS use with the age of asthma onset, and secondary hypotheses exploring the interaction of P30D ENDS use by sex and by race and ethnicity are in preparation.

## Conclusions

This cohort study found that adults who reported never using cigarettes, not having asthma or COPD, and P30D ENDS use at the first wave of participation had increased risk of asthma incidence at earlier ages in comparison with adults who reported no P30D ENDS use. Systematic efforts are needed to curb the health burden of asthma, including earlier disease screening. Modifying screening asthma guidelines to incorporate the P30 ENDS use may result in early detection of asthma and can lead to better symptom control, lower doses of medication, fewer adverse effects, and improved treatment outcomes.^52^ Prevention and cessation programs directed at ENDS use are needed to alleviate its impact on the age of asthma onset.

## Notes

Supplement 1. **eTable 1.** Participants Excluded or Included in the Primary Analysis Among Adults and Youths That Did Not Have Asthma or Chronic Obstructive Pulmonary Disease and Never Used Cigarettes in the PATH Study (2013-2021) at the First Wave of Participation **eTable 2.** Participants Excluded or Included in the Sensitivity Analysis Among Adults and Youth That Did Not Have Asthma or Chronic Obstructive Pulmonary Disease in the PATH Study (2013-2021) at the First Wave of Participation **eTable 3**. Sensitivity Analysis: Demographic and Measure Characteristics of Adults and Youths That Were Asthma/COPD Free in the PATH Study (2013-2021) at the First Wave of Participation **eTable 4.** Sensitivity Analysis: Crude and Adjusted Hazard Ratios (AHR) Examining the Association of Past 30-Day (P30D) ENDS Use on the Age of Asthma Onset Among Adults Who Were Asthma/COPD Free US at the First Wave of Participation in the PATH Study (2013-2021) (n = 31,393) **eTable 5.** Sensitivity Analysis: Crude and Adjusted Hazard Ratios (AHR) Examining the Association of Past 30-Day (P30D) ENDS Use on the Age of Asthma Onset Among Youth Who Did Not Have Asthma at the First Wave of Participation in the PATH Study (2013-2021) (n = 17,462) **eFigure.** Sensitivity Analysis: Cumulative Hazard Function for Age of Asthma Onset Among Participants Who Did Not Have Asthma or Chronic Obstructive Pulmonary Disease at the First Wave of Participation in the PATH Study (2013-2021)

Supplement 2.
Data Sharing Statement
