# Supplementary material for: Use of Electronic Nicotine Delivery Systems and Age of Asthma Onset Among US Adults and Youths
Source: JAMA Netw Open. 2024 May 17;7(5):e2410740. doi: 10.1001/jamanetworkopen.2024.10740 (PMC11102021; doi:10.1001/jamanetworkopen.2024.10740)
Supplement: Supplement 1. — eTable 1. Participants Excluded or Included in the Primary Analysis Among Adults and Youths That Did Not Have Asthma or Chronic Obstructive Pulmonary Disease and Never Used Cigarettes in the PATH Study (2013-2021) at the First Wave of Participation eTable 2. Participants Excluded or Included in the Sensitivity Analysis Among Adults and Youth That Did Not Have Asthma or Chronic Obstructive Pulmonary Disease in the PATH Study (2013-2021) at the First Wave of Participation eTable 3. Sensitivity Analysis: Demographic and Measure Characteristics of Adults and Youths That Were Asthma/COPD Free in the PATH Study (2013-2021) at the First Wave of Participation eTable 4. Sensitivity Analysis: Crude and Adjusted Hazard Ratios (AHR) Examining the Association of Past 30-Day (P30D) ENDS Use on the Age of Asthma Onset Among Adults Who Were Asthma/COPD Free US at the First Wave of Participation in the PATH Study (2013-2021) (n = 31,393) eTable 5. Sensitivity Analysis: Crude and Adjusted Hazard Ratios (AHR) Examining the Association of Past 30-Day (P30D) ENDS Use on the Age of Asthma Onset Among Youth Who Did Not Have Asthma at the First Wave of Participation in the PATH Study (2013-2021) (n = 17,462) eFigure. Sensitivity Analysis: Cumulative Hazard Function for Age of Asthma Onset Among Participants Who Did Not Have Asthma or Chronic Obstructive Pulmonary Disease at the First Wave of Participation in the PATH Study (2013-2021) [file jamanetwopen-e2410740-s001.pdf]

Supplemental Online Content

Pérez A, Valencia S, Jani PP, Harrell MB. Use of electronic nicotine delivery systems and the age of asthma onset among US adults and youths. *JAMA Netw Open*. 2024;7(5):e2410740.
doi:10.1001/jamanetworkopen.2024.10740

eTable 1. Participants Excluded or Included in the Primary Analysis Among Adults and Youths That Did Not Have Asthma or Chronic Obstructive Pulmonary Disease and Never Used Cigarettes in the PATH Study (2013-2021) at the First Wave of Participation.

eTable 2. Participants Excluded or Included in the Sensitivity Analysis Among Adults and Youths That Did Not Have Asthma or Chronic Obstructive Pulmonary Disease in the PATH Study (2013-2021) at the First Wave of Participation.

eTable 3. Sensitivity Analysis: Demographic and Measure Characteristics of Adults and Youths That Reported Not Having Asthma or Chronic Obstructive Pulmonary Disease in the PATH Study (2013-2021) at the First Wave of Participation.

eTable 4. Sensitivity Analysis: Association of Past 30-Day (P30D) ENDS Use With the Age of Asthma Onset Among the 31,393 Adults who Did Not Have Asthma or Chronic Obstructive Pulmonary Disease at the First Wave of Participation in the PATH Study, 2013-2021.

eTable 5. Sensitivity Analysis: Association of Past 30-Day (P30D) ENDS Use With the Age of Asthma Onset Among the 17,462 Youths Who Did Not Have Asthma at the First Wave of Participation in the PATH Study, 2013-2021.

eFigure. Sensitivity Analysis: Cumulative Hazard Function for Age of Asthma Onset Among Participants Who Did Not Have Asthma or Chronic Obstructive Pulmonary Disease at the First Wave of Participation in the PATH Study (2013-2021).

This supplemental material has been provided by the authors to give readers additional information about their work.

© 2024 Pérez A et al. *JAMA Network Open.*

eTable 1: Participants Excluded or Included in the Primary Analysis Among Adults and Youths That Did Not Have Asthma or Chronic Obstructive Pulmonary Disease and Never Used Cigarettes in the PATH Study (2013-2021) at the First Wave of Participation.

**Adults**			
Variable reported at the first wave of participation in the PATH[a] Study	Sample No.	Estimated national population No.	Weighted % (SE[b])
**Excluded participants**			
A doctor or other health professional said the participant had asthma	1,108	9,621,006	3.55 (0.13)
Age was missing	3	45,487	0.02 (0.01)
The answer to “doctor or other health professional said you had asthma?” was missing	39	472,621	0.17 (0.04)
Cigarette use status was missing	24	306,990	0.11 (0.03)
Past 30-day use of ENDS[c] was missing	17	280,672	0.10 (0.03)
Reported cigarette use	29,376	179,672,781	66.29 (0.58)
Reported COPD[d]	23	445,480	0.16 (0.04)
Reported COPD[d] and asthma	29	194,195	0.07 (0.02)
**Included participants**			
Asthma/COPD[d] free and who reported never use of cigarettes	7,766	80,006,590	29.52 (0.52)
Total	38,385	271,045,821	100
**Youths**			
**Excluded participants**			
A doctor or other health professional said participant had asthma	3,655	7,120,108	15.72 (0.27)
The answer to “doctor or other health professional said you had asthma?” was missing	92	215,983	0.48 (0.05)
Cigarette use status was missing	34	62,329	0.14 (0.03)
Past 30-day use of ENDS[c] was missing	94	188,572	0.42 (0.05)
Reported cigarette use	2,071	3,794,131	8.38 (0.26)
**Included participants**			
Asthma free and who reported never use of cigarettes	17,023	33,901,949	74.87 (0.37)
Total	22,969	45,283,072	100

a. The restricted file received disclosure to publish: 10/25/23-10/31/23. United States Department of Health and Human Services. National Institutes of Health. National Institute on Drug Abuse, and United States Department of Health and Human Services. Food and Drug Administration. Center for

© 2024 Pérez A et al. *JAMA Network Open.*

Tobacco Products. Population Assessment of Tobacco and Health (PATH) Study [United States] Restricted-Use Files. Inter-university Consortium for Political and Social Research [distributor], 2023-05-19. https://doi.org/10.3886/ICPSR36231.v36.
b. Any difference in response categories with the total sum of the estimated population size is due to rounding of decimals, SE=standard error.
c. ENDS include vapes, vaporizers, vape pens, hookah pens, electronic cigarettes, and/or electronic pipes.
d. COPD: Chronic obstructive pulmonary disease.

© 2024 Pérez A et al. *JAMA Network Open.*

eTable 2: Participants Excluded or Included in the Sensitivity Analysis Among Adults and Youths That Did Not Have Asthma or Chronic Obstructive Pulmonary Disease in the PATH Study (2013-2021) at the First Wave of Participation.

Adults			
Variable reported at the first wave of participation in the PATH[a] Study	Sample No.	Estimated national population No.	Weighted % (SE[b])
**Excluded participants**			
A doctor or other health professional said the participant had asthma	4,891	30,159,529	11.13 (0.20)
Age was missing	8	114,572	0.04 (0.02)
The answer to "doctor or other health professional said you had asthma?" was missing	174	1,353,042	0.50 (0.06)
Body Mass Index was missing	576	5,003,269	1.85 (0.11)
Ever use of any cigar was missing	104	1,210,233	0.45 (0.06)
Ever use of cigarettes was missing	11	146,479	0.05 (0.03)
Ever use of hookah was missing	40	358,107	0.13 (0.03)
Past 30-day use of ENDS[c] was missing	55	437,926	0.16 (0.03)
Past 30-day use of smokeless tobacco was missing	235	1,306,647	0.48 (0.04)
Reported COPD[d]	898	5,920,022	2.18 (0.11)
**Included participants**			
Asthma/COPD[d] free and who reported combustible[e] tobacco product use	25,983	158,607,112	58.52 (0.56)
Asthma/COPD[d] free and who reported no combustible[e] tobacco product use	5,410	66,428,885	24.51 (0.54)
Total	38,385	271,045,821	100
**Youths**			
**Excluded participants**			
A doctor or other health professional said the participant had asthma	4,072	7,865,429	17.37 (0.28)
The answer to "doctor or other health professional said you had asthma?" was missing	112	254,187	0.56 (0.06)
Body Mass Index was missing	766	1,539,851	3.40 (0.13)
Ever use of any cigar was missing	193	381,377	0.84 (0.07)
Ever use of cigarettes was missing	14	27,670	0.06 (0.02)
Ever use of hookah was missing	39	72,462	0.16 (0.03)
Past 30-day use of ENDS[c] was missing	88	181,738	0.40 (0.05)
Past 30-day use of smokeless tobacco was missing	176	318,126	0.70 (0.06)

© 2024 Pérez A et al. *JAMA Network Open.*

Race/ethnicity was missing	35	76,861	0.17 (0.04)
Sex was missing	12	26,446	0.06 (0.02)
**Included participants**			
Asthma free and who reported combustible[e] tobacco product use	2,023	3,737,889	8.25 (0.25)
Asthma free and who reported no combustible[e] tobacco product use	15,439	30,801,034	68.02 (0.41)
Total	22,969	45,283,071	100

a. The restricted file received disclosure to publish: 07/21/23-07/27/23. United States Department of Health and Human Services. National Institutes of Health. National Institute on Drug Abuse, and United States Department of Health and Human Services. Food and Drug Administration. Center for Tobacco Products. Population Assessment of Tobacco and Health (PATH) Study [United States] Restricted-Use Files. Inter-university Consortium for Political and Social Research [distributor], 2023-05-19. https://doi.org/10.3886/ICPSR36231.v36.
b. Any difference in response categories with the total sum of the estimated population size is due to rounding of decimals, SE=standard error.
c. ENDS include vapes, vaporizers, vape pens, hookah pens, electronic cigarettes, and/or electronic pipes.
d. COPD: Chronic obstructive pulmonary disease.
e. Cigarettes, hookah, and any cigars (cigarillos, filtered cigars, traditional cigars).

© 2024 Pérez A et al. *JAMA Network Open.*

Supplemental Methods from this point forward included all PATH adult and youth participants who were asthma/COPD free at the first wave of participation. There were 31,393 adults and 17,462 youth, representing 225 million and 34.5 million USA adults and youth, respectively.

eTable 3: Sensitivity Analysis: Demographic and Measure Characteristics of Adults and Youths That Reported Not Having Asthma or Chronic Obstructive Pulmonary Disease in the PATH Study (2013-2021) at the First Wave of Participation.

**Variable reported at the first wave of participation in the PATH[a] Study**		Adults			Youths		
		Sample No. (n=31,393)[b]	Estimated national population No. (N=225,035,997)[b]	Weighted % (SE)[b]	Sample No. (n=17,462)[b]	Estimated national population No. (N=34,538,924)[b]	Weighted % (SE)[b]
Wave of entry into the PATH[a] Study							
1 (2013-2014)		26,472	196,646,267	87.38 (0.12)	10,533	19,257,224	55.76 (0.22)
2 (2014-2015)		NA	NA	NA	1,505	3,002,504	8.69 (0.14)
3 (2015-2016)		NA	NA	NA	1,510	3,148,646	9.11 (0.17)
4 (2016-2018)		4,921	28,389,730	12.62 (0.12)	1,284	2,362,400	6.84 (0.11)
4.5(2017-2018)		NA	NA	NA	1,305	3,199,064	9.26 (0.16)
5 (2018-2019)		NA	NA	NA	1,264	3,332,179	9.65 (0.16)
5.5 (2019-2020)		NA	NA	NA	40	144,163	0.42 (0.06)
6 (2021-2021)		NA	NA	NA	21	92,743	0.27 (0.06)
Age [weighted mean (SE)]		45.96 (0.08)			13.45 (0.01)		
Sex	Female	15,036	113,779,453	50.56 (0.18)	8,693	17,340,229	50.20 (0.18)
	Male	16,357	111,256,544	49.44 (0.18)	8,769	17,198,694	49.80 (0.18)
Race and ethnicity	Hispanic	5,636	34,559,656	15.36 (0.12)	4,875	7,872,661	22.79 (0.17)
	Non-Hispanic black	4,597	26,968,522	11.98 (0.10)	2,143	4,323,080	12.52 (0.18)
	Non-Hispanic white	18,730	145,402,980	64.61 (0.18)	8,817	18,846,120	54.56 (0.26)
	Other[c]	2,430	18,104,839	8.05 (0.10)	1,627	3,497,062	10.13 (0.17)
Education level[d]	Less than high school	5,987	35,657,324	15.85 (0.12)	3,356	5,728,473	16.59 (0.46)
	High school/GED[e]	7,482	54,821,184	24.36 (0.15)	3,184	6,000,189	17.37 (0.37)

© 2024 Pérez A et al. *JAMA Network Open.*

	Some college/associate degree	11,013	69,700,717	30.97 (0.15)	6,822	14,026,835	40.61 (0.53)
	Bachelor's degree or higher	6,911	64,856,772	28.82 (0.15)	3,922	8,442,331	24.44 (0.73)
	Did not respond	NA	NA	NA	178	341,096	0.99 (0.07)
Weight status categories[f]	Underweight	882	5,429,549	2.41 (0.13)	901	1,845,658	5.34 (0.18)
	Healthy weight	11,647	77,317,287	34.36 (0.39)	11,120	22,406,241	64.87 (0.41)
	Overweight	10,044	76,713,033	34.10 (0.37)	2,915	5,596,372	16.20 (0.29)
	Obesity (class 1 adult)	5,289	39,649,039	17.62 (0.34)	1,726	3,232,482	9.36 (0.23)
	Obesity (class 2 adult)	3,531	25,927,088	11.52 (0.27)			
	Severe obesity (youth)	NA	NA	NA	800	1,458,170	4.22 (0.17)
Past 30 day (P30D) combustible[g] tobacco product (TP) use and P30D ENDS[h] use status							
Never use of combustible[g] TP and no P30D ENDS[h] use		5,355	66,284,781	29.46 (0.63)	15,394	30,704,652	88.90 (0.34)
Never use of combustible[g] TP and P30D ENDS[h] use		62	179,569	0.08 (0.01)	59	118,999	0.34 (0.05)
Former use of any combustible[g] TP and no P30D ENDS[h] use		10,306	100,315,196	44.58 (0.51)	1,189	2,203,625	6.38 (0.22)
Former use of any combustible[g] TP and P30D ENDS[h] use		618	2,245,110	1.00 (0.05)	100	195,912	0.57 (0.07)
P30D use of any combustible[g] TP and no P30D ENDS[h] use		11,514	43,531,300	19.34 (0.25)	517	946,147	2.74 (0.15)
P30D use of any combustible[g] TP and P30D ENDS[h] use		3,538	12,480,040	5.55 (0.13)	203	369,590	1.07 (0.08)
Binge drinking	No	27,532	207,576,522	92.24 (0.19)	17,088	33,819,275	97.92 (0.12)
	Yes	3,790	16,892,984	7.51 (0.18)	269	503,807	1.46 (0.09)
	Missing	71	566,491	0.25 (0.05)	105	215,842	0.62 (0.07)
Ever marijuana use	No	15,606	141,231,951	62.76 (0.56)	15,916	31,695,481	91.77 (0.28)
	Yes	15,627	82,755,175	36.77 (0.55)	1,435	2,611,575	7.56 (0.26)
	Missing	160	1,048,871	0.47 (0.05)	111	231,868	0.67 (0.08)
Any person who uses tobacco	No	20,085	168,711,544	74.97 (0.43)	11,508	23,140,729	67.00 (0.69)
	Yes	11,224	55,731,240	24.77 (0.43)	5,771	11,064,378	32.03 (0.69)

© 2024 Pérez A et al. *JAMA Network Open.*

product(s) present at home	Missing	84	593,213	0.26 (0.04)	183	333,816	0.97 (0.08)
Rules at home about tobacco product use (anywhere/ sometimes/anytime)	Never allowed	18,582	158,273,107	70.33 (0.49)	12,401	24,643,754	71.35 (0.59)
	Allowed	12,778	66,485,587	29.54 (0.49)	4,915	9,602,465	27.80 (0.58)
	Missing	33	277,303	0.12 (0.03)	146	292,705	0.85 (0.06)

a. The restricted file received disclosure to publish: 07/21/23-04/10/24. United States Department of Health and Human Services. National Institutes of Health. National Institute on Drug Abuse, and United States Department of Health and Human Services. Food and Drug Administration. Center for Tobacco Products. Population Assessment of Tobacco and Health (PATH) Study [United States] Restricted-Use Files. Inter-university Consortium for Political and Social Research [distributor], 2023-05-19. https://doi.org/10.3886/ICPSR36231.v36.

b. n=sample size, N=estimated population size, any difference in categories of response with the total sum of the estimated population size is due to rounding of decimals, SE=standard error.

c. Other included Non-Hispanic Asian, multiracial, and any other race or ethnicity not otherwise specified.

d. Highest level of education attained by participants for adults or highest level of education attained by parents for youths.

e. GED: General Educational Development Test.

f. Weight status was computed as follows: Adult body mass index (BMI) was calculated as weight in kilograms divided by height in meters squared. Adults with a BMI score less than 18.5 were classified as underweight, 18.5 to 25 as healthy weight, 25 to 30 as overweight, 30 to 35 as obesity class 1, and greater than 35 as obesity class 2. Youth BMI was calculated using the Centers for Disease Control BMI-for-age percentile growth charts that utilize the child’s age, height, and weight to determine percentiles. Youths who had a BMI less than the 5th percentile were classified as underweight, between the 5th and less than the 85th percentile as healthy weight, between the 85th and less than the 95th percentile as overweight, the 95th percentile or greater as obese, and 120% of the 95th percentile or greater or BMI of 35kg/m2 or greater as severe obesity.

g. Cigarettes, hookah, and any cigars (cigarillos, filtered cigars, traditional cigars).

h. ENDS include vapes, vaporizers, vape pens, hookah pens, electronic cigarettes, and/or electronic pipes.

© 2024 Pérez A et al. *JAMA Network Open.*

eTable 4: Sensitivity Analysis: Association of Past 30-Day (P30D) ENDS Use With the Age of Asthma Onset Among the 31,393 Adults who Did Not Have Asthma or Chronic Obstructive Pulmonary Disease at the First Wave of Participation in the PATH Study, 2013-2021.

**Variables measured at the first wave of participation in the PATH[a] Study**		**Crude Association**	**Model 1**	**Model 2**
		**HR (95% CI)**	**AHR (95% CI)**	**AHR (95% CI)**
Past 30 day (P30D) combustible[b] tobacco product (TP) use and P30D ENDS[c] use status.				
Never use of combustible[b] TP and no P30D ENDS[c] use		1	1	1
Never use of combustible[b] TP and P30D ENDS[c] use		2.86 (0.36-22.82)	1.42 (0.14-14.59)	1.45 (0.14-15.04)
Former use of any combustible[b] TP and no P30D ENDS[c] use		0.93 (0.72-1.20)	0.92 (0.70-1.22)	0.93 (0.70-1.22)
Former use of any combustible[b] TP and P30D ENDS[c] use		1.22 (0.65-2.29)	0.95 (0.49-1.84)	0.97 (0.51-1.84)
P30D use of any combustible[b] TP and no P30D ENDS[c] use		**2.11 (1.64-2.72)**	**1.67 (1.22-2.30)**	**1.69 (1.25-2.29)**
P30D use of any combustible[b] TP and P30D ENDS[c] use		**3.36 (2.55-4.44)**	**2.40 (1.71-3.38)**	**2.43 (1.77-3.34)**
Sex	Male	1	1	1
	Female	**1.64 (1.41-1.90)**	**1.82 (1.54-2.16)**	**1.82 (1.54-2.16)**
Race/ethnicity	Hispanic	**1.34 (1.07-1.68)**	**1.38 (1.08-1.77)**	**1.38 (1.07-1.77)**
	Non-Hispanic black	**1.83 (1.49-2.25)**	**1.53 (1.24-1.88)**	**1.53 (1.24-1.88)**
	Non-Hispanic white	1	1	1
	Other[d]	**1.53 (1.11-2.11)**	**1.66 (1.22-2.26)**	**1.66 (1.22-2.25)**
Education[e]	Some college/associate degree	1	1	1
	Less than high school	0.98 (0.82-1.18)	0.87 (0.71-1.06)	1.09 (0.85-1.39)
	High school/GED[f]	**0.80 (0.63-1.02)**	0.81 (0.64-1.02)	1.02 (0.77-1.33)
	Bachelor degree or higher	**0.61 (0.47-0.78)**	0.80 (0.62-1.03)	1.26 (0.97-1.62)
Weight status categories[g]	Healthy weight	1	1	1
	Underweight	1.14 (0.72-1.80)	0.99 (0.64-1.52)	0.99 (0.64-1.52)
	Overweight	1.09 (0.89-1.33)	1.18 (0.96-1.45)	1.18 (0.96-1.45)
	Obesity (class 1)	**1.27 (1.03-1.57)**	**1.32 (1.07-1.63)**	**1.32 (1.07-1.64)**
	Obesity (class 2)	**2.36 (1.85-3.01)**	**2.25 (1.75-2.88)**	**2.25 (1.76-2.88)**
Binge drinking[h]	No	1	1	1
	Yes	**2.24 (1.70-2.94)**	**1.57 (1.20-2.06)**	**1.57 (1.20-2.05)**
Ever marijuana use[h]	No	1	1	1

© 2024 Pérez A et al. *JAMA Network Open.*

	Yes	**1.78 (1.47-2.15)**	**1.59 (1.27-1.99)**	**1.60 (1.27-2.00)**
Any person who uses tobacco product(s) present at home[h]	No	1	1	1
	Yes	**2.06 (1.74-2.45)**	**1.41 (1.20-1.67)**	**1.42 (1.20-1.69)**
Rules at home about tobacco product use[h] (anywhere or sometimes/anytime)	Never allowed	1	1	NA
	Allowed	**1.56 (1.31-1.86)**	1.03 (0.85-1.25)	NA

a. The restricted file received disclosure to publish: 07/20/23-11/13/23. United States Department of Health and Human Services. National Institutes of Health. National Institute on Drug Abuse, and United States Department of Health and Human Services. Food and Drug Administration. Center for Tobacco Products. Population Assessment of Tobacco and Health (PATH) Study [United States] Restricted-Use Files. Inter-university Consortium for Political and Social Research [distributor], 2023-05-19. https://doi.org/10.3886/ICPSR36231.v36.
b. Cigarettes, hookah, and any cigars (cigarillos, filtered cigars, traditional cigars).
c. ENDS include vapes, vaporizers, vape pens, hookah pens, electronic cigarettes, and/or electronic pipes.
d. Other included Non-Hispanic Asian, multiracial, and any other race or ethnicity not otherwise specified.
e. Highest level of education attained by participants for adults or highest level of education attained by parents for youths.
f. GED: General Educational Development Test.
g. Weight status was computed as follows: Adult body mass index (BMI) was calculated as weight in kilograms divided by height in meters squared. Adults with a BMI score less than 18.5 were classified as underweight, 18.5 to 25 as healthy weight, 25 to 30 as overweight, 30 to 35 as obesity class 1, and greater than 35 as obesity class 2.
h. The did not respond category is accounted for in the model but the HR is not shown.

© 2024 Pérez A et al. *JAMA Network Open.*

eTable 5: Sensitivity Analysis: Association of Past 30-Day (P30D) ENDS Use With the Age of Asthma Onset Among the 17,462 Youths Who Did Not Have Asthma at the First Wave of Participation in the PATH Study, 2013-2021.

**Variables measured at the first wave of participation in the PATH[a] Study**		**Crude Association**	**Model 1**	**Model 2**
		**HR (95% CI)**	**AHR (95% CI)**	**AHR (95% CI)**
Past 30 day (P30D) combustible[b] tobacco product (TP) use and P30D ENDS[c] use status				
Never use of combustible[b] TP and no P30D ENDS[c] use		1	1	1
Never use of combustible[b] TP and P30D ENDS[c] use		0.66 (0.10-4.24)	0.70 (0.10-4.83)	0.68 (0.10-4.65)
Former use of any combustible[b] TP and no P30D ENDS[c] use		0.77 (0.60-1.03)	0.81 (0.58-1.12)	0.80 (0.57-1.12)
Former use of any combustible[b] TP and P30D ENDS[c] use		1.46 (0.74-2.89)	1.68 (0.83-3.38)	1.64 (0.81-3.33)
P30D use of any combustible[b] TP and no P30D ENDS[c] use		0.88 (0.63-1.23)	0.95 (0.64-1.41)	0.91 (0.61-1.34)
P30D use of any combustible[b] TP and P30D ENDS[c] use		1.02 (0.63-1.65)	1.17 (0.68-2.02)	1.09 (0.63-1.90)
Sex[d]	Male	1	1	1
	Female	**1.43 (1.26-1.64)**	**1.45 (1.26-1.66)**	**1.44 (1.26-1.65)**
Race/ethnicity[d]	Hispanic	0.92 (0.80-1.06)	0.94 (0.79-1.11)	0.93 (0.79-1.09)
	Non-Hispanic black	**1.26 (1.03-1.54)**	**1.23 (1.01-1.51)**	**1.24 (1.01-1.52)**
	Non-Hispanic white	1	1	1
	Other[e]	1.06 (0.86-1.32)	1.05 (0.85-1.32)	1.06 (0.85-1.32)
Parent's education[d,f]	Some college/ associates degree	1	1	1
	Less than high school	1.03 (0.83-1.28)	0.97 (0.74-1.26)	0.98 (0.75-1.27)
	High school/GED[g]	**0.72 (0.58-0.91)**	**0.69 (0.52-0.86)**	**0.67 (0.52-0.87)**
	Bachelor's degree or higher	0.99 (0.80-1.23)	0.93 (0.74-1.17)	0.94 (0.74-1.18)
Weight status categories[h]	Healthy weight	1	1	1
	Underweight	0.82 (0.56-1.19)	0.87 (0.60-1.26)	0.88 (0.61-1.27)
	Overweight	1.02 (0.82-1.27)	1.05 (0.84-1.31)	1.05 (0.84-1.31)
	Obesity	1.20 (0.94-1.55)	1.26 (0.98-1.63)	1.27 (0.98-1.63)
	Severe obesity	**1.40 (1.00-1.98)**	1.42 (1.00-2.02)	1.43 (1.00-2.03)
Binge drinking[d]	No	1	1	NA
	Yes	0.64 (0.35-1.18)	0.65 (0.34-1.23)	NA
Ever marijuana use[d]	No	1	1	1

© 2024 Pérez A et al. *JAMA Network Open.*

	Yes	0.87 (0.71-1.07)	0.92 (0.67-1.27)	0.89 (0.64-1.24)
Any person who uses tobacco product(s) presence at home[d]	No	1	1	1
	Yes	**1.20 (1.04-1.40)**	**1.23 (1.02-1.48)**	**1.24 (1.05-1.47)**
Rules at home about tobacco product use[d] (anywhere or sometimes/anytime)	Never allowed	1	1	NA
	Allowed	1.10 (0.96-1.27)	1.03 (0.87-1.22)	NA

a. The restricted file received disclosure to publish: 07/20/2023-04/10/24. United States Department of Health and Human Services. National Institutes of Health. National Institute on Drug Abuse, and United States Department of Health and Human Services. Food and Drug Administration. Center for Tobacco Products. Population Assessment of Tobacco and Health (PATH) Study [United States] Restricted-Use Files. Inter-university Consortium for Political and Social Research [distributor], 2023-05-19. https://doi.org/10.3886/ICPSR36231.v36.

b. Cigarettes, hookah, and any cigars (cigarillos, filtered cigars, traditional cigars).

c. ENDS include vapes, vaporizers, vape pens, hookah pens, electronic cigarettes, and/or electronic pipes.

d. The did not respond category is accounted for in the model but the HR is not shown.

e. Other included Non-Hispanic Asian, multiracial, and any other race or ethnicity not otherwise specified.

f. Highest level of education attained by participants for adults or highest level of education attained by parents for youths.

g. GED: General Educational Development Test.

h. Weight status was computed as follows: Youth BMI was calculated using the Centers for Disease Control BMI-for-age percentile growth charts that utilize the child's age, height, and weight to determine percentiles. Youths who had a BMI less than the 5th percentile were classified as underweight, between the 5th and less than the 85th percentile as healthy weight, between the 85th and less than the 95th percentile as overweight, the 95th percentile or greater as obese, and 120% of the 95th percentile or greater or BMI of 35kg/m2 or greater as severe obesity.

© 2024 Pérez A et al. *JAMA Network Open.*

eFigure: Sensitivity Analysis: Cumulative Hazard Function for Age of Asthma Onset Among Participants Who Did Not Have Asthma or Chronic Obstructive Pulmonary Disease at the First Wave of Participation in the PATH Study (2013-2021).

Panel (a) Adults

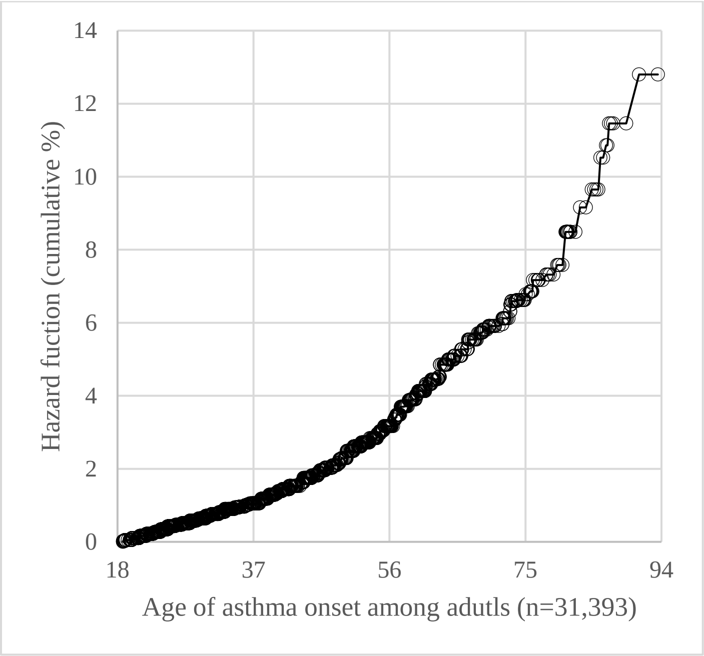

Panel (b) Youths

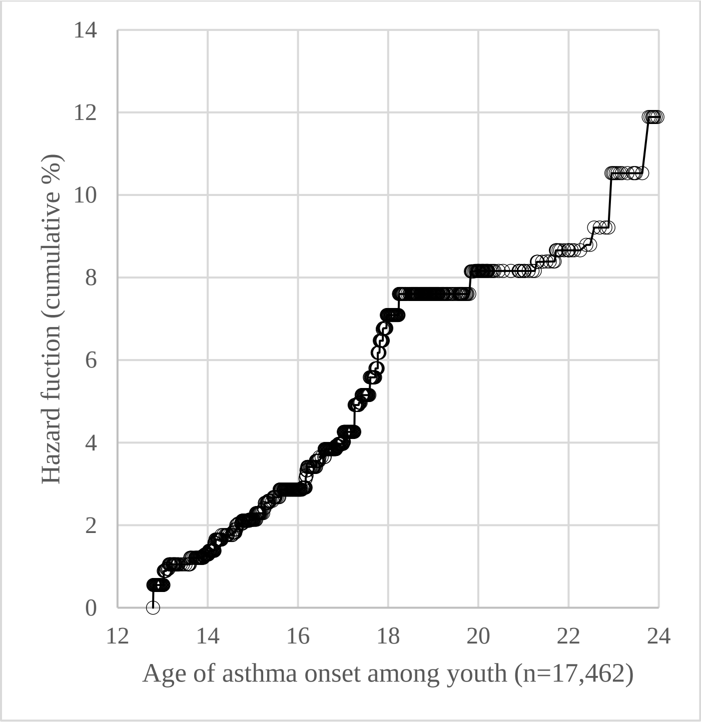

© 2024 Pérez A et al. *JAMA Network Open.*
