# Supplementary material for: Use of Electronic Nicotine Delivery Systems and Age of Asthma Onset Among US Adults and Youths
Source: JAMA Netw Open. 2024 May 17;7(5):e2410740. doi: 10.1001/jamanetworkopen.2024.10740 (PMC11102021; doi:10.1001/jamanetworkopen.2024.10740)
Supplement: Supplement 2. — Data Sharing Statement [file jamanetwopen-e2410740-s002.pdf]

Data Sharing Statement

Pérez. Use of Electronic Nicotine Delivery Systems and Age of Asthma Onset Among US Adults and Youths. *JAMA Netw Open*. Published May 10, 2024.
doi:10.1001/jamanetworkopen.2024.10740

Data
Data available: No

Additional Information
Explanation for why data not available: All the data from waves 1-6 are available from the Population Assessment of Tobacco and Health (PATH) Study [United States] Restricted-Use Files. Inter-university Consortium for Political and Social Research [distributor], 2020-06-24. https://doi.org/10.3886/ICPSR36231.v25. Data are available from https://www.icpsr.umich.edu/icpsrweb/NAHDAP/studies/36498/datadocumentation#.
